# Childhood Obesity: Today and Tomorrow's Health Challenge

**DOI:** 10.1155/2013/208392

**Published:** 2013-11-12

**Authors:** Roya Kelishadi, Sarah D. de Ferranti, Reza Majdzadeh, Jennifer A. O'Dea, Ajay K. Gupta, Khosrow Adeli

**Affiliations:** ^1^Pediatrics, Child Growth and Development Research Center, Isfahan University of Medical Sciences, Isfahan 81676-36954, Iran; ^2^Pediatric Cardiology, Preventive Cardiology Department, Children's Hospital, Boston, MA, USA; ^3^Epidemiology, School of Public Health, and Knowledge Utilization Research Center, Tehran University of Medical Sciences, Tehran, Iran; ^4^Faculty of Education & Social Work, The University of Sydney, Sydney, Australia; ^5^International Centre for Circulatory Health, National Heart and Lung Institute, Imperial College, London, UK; ^6^Clinical Biochemistry, The Hospital for Sick Children, University of Toronto, Toronto, Canada

In the current special issue different aspects of childhood obesity and metabolic syndrome (MetS) are being discussed. Childhood obesity is becoming an emerging health problem at individual and public health level. The problem is no more limited to high-income countries and is rapidly growing in low- and middle-income countries [1, 2]. Its early- and late-onset complications warrant studying more about this important issue [3].

Nowadays, the obesity epidemic and its associated complications as MetS, type 2 diabetes mellitus, cardiovascular diseases, and nonalcoholic fatty liver disease are considered as a global health problem. Its etiology is multifactorial consisting of the interaction between genetics and environmental factors, lifestyle behaviors, and sociodemographic background. Risk factors of chronic diseases originate from early life and are tracked from childhood to adulthood [4]. Therefore, prevention, screening, and early control of excess weight and related risk factors might help tailoring intervention strategies against the excess burden of noncommunicable diseases (NCDs) [5].

Health policies for prevention and control of childhood obesity have to be made by developing action-oriented intervention strategies, mainly by community participatory activities in each population. The role of families, notably parents and grandparents, should be highlighted in this regard, and family centered interventions should be encouraged [6].

The rapidly increasing trend of childhood obesity and MetS is alarming and provides information for policymakers and health care providers for interventional preventive programs. Public health and clinical aspects should be considered for evidence-based solutions to the current challenges in health promotion and disease prevention.

The current issue presents papers that seek to define the determinants as well as prevention and treatment of childhood obesity and its various complications in various potential topics including recent developments on the etiology of childhood obesity and metabolic syndrome, reports on determinants of overweight/obesity in the pediatric age group in different countries, action-oriented preventive measures against excess weight in children, and reports on short-term and long-term complications of childhood obesity, as well as therapeutic modalities in this field.

*Roya Kelishadi* *Roya Kelishadi* *Sarah D. de Ferranti* *Sarah D. de Ferranti* *Reza Majdzadeh* *Reza Majdzadeh* *Jennifer A. O'Dea* *Jennifer A. O'Dea* *Ajay K. Gupta* *Ajay K. Gupta* *Khosrow Adeli* *Khosrow Adeli*

## References

[1] Finucane MM, Stevens GA, Cowan MJ (2011). National, regional, and global trends in body-mass index since 1980: systematic analysis of health examination surveys and epidemiological studies with 960 country-years and 9*·*1 million participants. *The Lancet*.

[2] Gupta N, Shah P, Nayyar S, Misra A (2013). Childhood obesity and the metabolic syndrome in developing countries. *The Indian Journal of Pediatrics *.

[3] Weiss R, Caprio S (2005). The metabolic consequences of childhood obesity. *Best Practice and Research Clinical Endocrinology and Metabolism*.

[4] Venn AJ, Thomson RJ, Schmidt MD (2007). Overweight and obesity from childhood to adulthood: a follow-up of participants in the 1985 Australian Schools Health and Fitness Survey. *Medical Journal of Australia*.

[5] Yach D, Hawkes C, Gould CL, Hofman KJ (2004). The global burden of chronic diseases: overcoming impediments to prevention and control. *The Journal of the American Medical Association*.

[6] Esfarjani F, Khalafi M, Mohammadi F (2013). Family-based intervention for controlling childhood obesity: an experience among Iranian children. *International Journal of Preventive Medicine*.

